# Engineering of CYP76AH15 can improve activity and specificity towards forskolin biosynthesis in yeast

**DOI:** 10.1186/s12934-018-1027-3

**Published:** 2018-11-19

**Authors:** Victor Forman, Niels Bjerg-Jensen, Jane D. Dyekjær, Birger Lindberg Møller, Irini Pateraki

**Affiliations:** 1 Plant Biochemistry Laboratory, Department of Plant and Environmental Sciences, University of Copenhagen, Thorvaldsensvej 40, 1871 Frederiksberg C, Denmark; 2 Evolva A/S, Copenhagen, Denmark; 3 bioSYNergy, Center for Synthetic Biology, 1871 Frederiksberg C, Denmark; 4 VILLUM, Research Center for Plant Plasticity, 1871 Frederiksberg C, Denmark

**Keywords:** Terpenoids, Diterpenes, Forskolin, CYP76AH, Metabolic engineering, Site-directed mutagenesis, CYPs, Cytochrome P450s, *Saccharomyces cerevisiae*

## Abstract

**Background:**

Forskolin is a high-value diterpenoid produced exclusively by the Lamiaceae plant *Coleus forskohlii.* Today forskolin is used pharmaceutically for its adenyl-cyclase activating properties. The limited availability of pure  forskolin is currently hindering its full utilization, thus a new environmentally friendly, scalable and sustainable strategy is needed for forskolin production. Recently, the entire biosynthetic pathway leading to forskolin was elucidated. The key steps of the pathway are catalyzed by cytochrome P450 enzymes (CYPs), which have been shown to be the limiting steps of the pathway. Here we study whether protein engineering of the substrate recognition sites (SRSs) of CYPs can improve their efficiency towards forskolin biosynthesis in yeast.

**Results:**

As a proof of concept, we engineered the enzyme responsible for the first putative oxygenation step of the forskolin pathway: the conversion of 13*R*-manoyl oxide to 11-oxo-13*R*-manoyl oxide, catalyzed by the CYP76AH15. Four CYP76AH15 variants—engineered in the SRS regions—yielded at least a twofold increase of 11-oxo-13*R*-manoyl oxide when expressed in yeast cells grown in microtiter plates. The highest titers (5.6-fold increase) were observed with the variant A99I, mutated in the SRS1 region. Double or triple CYP76AH15 mutant variants resulted in additional enzymes with optimized performances. Moreover, in planta CYP76AH15 can synthesize ferruginol from miltiradiene. In this work, we showed that the mutants affecting 11-oxo-13*R*-manoyl oxide synthesis, do not affect ferruginol production, and vice versa.

The best performing variant, A99I, was utilized to reconstruct the forskolin biosynthetic pathway in yeast cells. Although these strains showed increased 11-oxo-manoyl oxide production and higher accumulation of other pathway intermediates compared to the native CYP76AH15, lower production of forskolin was observed.

**Conclusions:**

As demonstrated for CYP76AH15, site-directed mutagenesis of SRS regions of plant CYPs may be an efficient and targeted approach to increase the performance of these enzymes. Although in this work we have managed to achieve higher efficiency and specificity of the first CYP of the pathway, further work is necessary in order to increase the overall production of forskolin in yeast cells.

**Electronic supplementary material:**

The online version of this article (10.1186/s12934-018-1027-3) contains supplementary material, which is available to authorized users.

## Background

Many diterpenoids from the Lamiaceae (or mint) plant family are high-value compounds that have been used extensively in the food, medicine and fragrance industries. Isolation of pure diterpenoids from native plants is challenging due to low amounts, difficult separation from structurally alike compounds, and yield fluctuations because of environmental factors. Forskolin is a labdane-diterpenoid produced exclusively from the Ayurvedic Lamiaceae plant *Coleus forskohlii.* The medicinal properties attributed to forskolin, from body mass control to glaucoma and heart failure treatment, are based on its ability to activate the enzyme adenyl-cyclase, which subsequently increases the cellular cAMP levels [1–3]. As no enantioselective chemical synthesis has been achieved [4], a potential alternative, sustainable and readily scalable way to produce forskolin would be to use biotechnological production systems whereby the biosynthetic pathway is reconstructed in a heterologous host. Such an approach requires not only the knowledge of the biosynthetic steps and enzymes, but also metabolic optimization of the pathway and the selected host, to reach industrially relevant production titers.

The biosynthetic pathway of forskolin was recently elucidated [5], enabling the development of biotechnological means for the production of this pharmaceutical. A *Saccharomyces cerevisiae* (yeast) strain expressing the entire forskolin biosynthetic pathway was able to produce 40 mg of forskolin per liter of yeast culture using fed batch fermentation [5]. Although the titers obtained were significant, it was still far from possible industrial applications. Nevertheless, the results obtained from these first experiments indicated the first promising points where the pathway could be optimize to increase forskolin production in yeast.

Forskolin is produced in the root cork cells of *C. forskohlii*, from GGPP (geranylgeranyl diphosphate), which is the building block of most diterpenoids. GGPP is converted to 13*R*-manoyl oxide, the cyclic diterpenoid precursor of forskolin [6], by a pair of soluble plastidial diterpene synthase enzymes (*Cf*TPS2 and *Cf*TPS3). Both diterpene synthases (diTPSs) were highly expressed in the root cork cells of *C. forskohlii,* where forskolin accumulates. *Cf*TPS1 is an additional highly expressed root cork specific diTPS that in combination with *Cf*TPS3 gives rise to the formation of miltiradiene, a precursor of a different group of diterpenoids found to accumulate in the same tissue. Miltiradiene is a precursor for a number of bioactive diterpenoids, like carnosic acid and tanshinones found in other Lamiaceae species [7, 8].

**Fig. 1:**
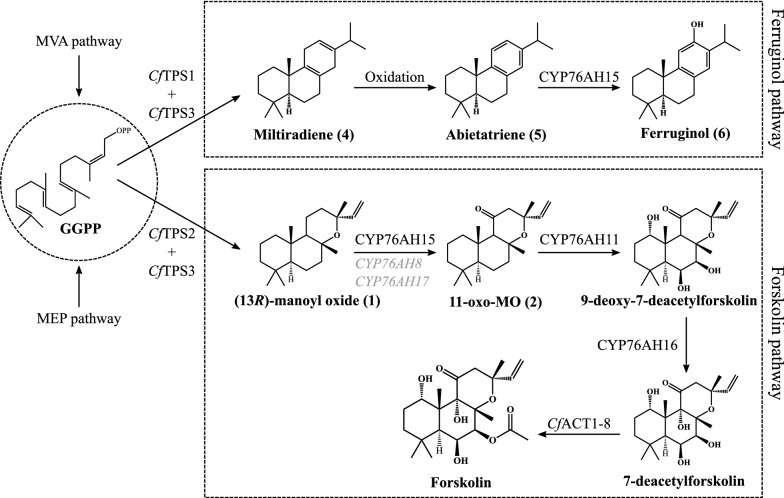
Diterpene biosynthetic pathways from the universal precursor geranylgeranyl diphosphate (GGPP) towards forskolin and ferruginol, compounds present in the root cork cells of *Coleus forskohlii*. The common diterpene precursor GGPP is formed by four C_5_ units from the plastid based 2-*C*-methyl-d-erythritol 4-phosphate pathway (MEP). GGPP is cyclized by the class II diterpene synthase (diTPS), *Cf*TPS1, to (+)-copalyl-diphosphate and further to miltiradiene by class I diTPS, *Cf*TPS3. Miltiradiene can undergo non-enzymatic oxidation into abietatriene, which is hydroxylated to ferruginol by CYP76AH15. The forskolin precursor 13*R*-manoyl oxide is formed from GGPP through (+)-8-hydroxy-copalyl diphosphate by the class II diTPS, *Cf*TPS2, and further to 13*R*-manoyl oxide by the class I diTPS, *Cf*TPS3. 13*R*-manoyl oxide is further converted by CYP76AH15 to 11-oxo-13*R*-manoyl oxide, which is further converted to 9-deoxy-7-deacetylforskolin by the multifunctional CYP76AH11 and to deacetyl-forskolin by CYP76AH16. Finally, deacetyl-forskolin is acetylated at carbon 7 by *Cf*ACT1-8 to forskolin

13*R*-Manoyl oxide is oxidatively decorated by three cytochrome P450 enzymes (CYPs), all belonging to the recently discovered Lamiaceae specific CYP76AH subfamily, resulting in the formation of deacetyl-forskolin. All forskolin-related CYP76AHs were found to be highly expressed in the root cork cells. The last step comprising acetylation of deacetylforskolin to forskolin was found to be catalyzed by the *Cf*ACT1-8 acetyltransferase (Fig. 1), a member of the BAHD family [5]. The functional biosynthetic pathway of forskolin was efficiently reconstituted in engineered *S. cerevisiae,* adding to the compilation of cases showing yeast as an efficient host platform for diterpenoid production [5, 7, 9, 10]. However, in this yeast strain high levels of 13*R*-manoyl oxide and CYP intermediates accumulated, indicating that the efficiency or specificity of the CYP catalysed reactions were compromising the attainment of increased titers of forskolin.

Plant CYPs are heme-binding microsomal enzymes typically bound to the endoplasmic reticulum (ER) membrane by an N-terminal peptide-anchor, with the catalytic domain of the enzyme exposed to the cytosol [11]. CYPs are unique catalysts as they can catalyze oxygenations of non-activated C-atom bonds under mild conditions [12]. Targeted site-directed mutagenesis of membrane-bound plant CYPs to increase or change their catalytic properties is challenging due to the limited number of available CYP's  crystal structures; moreover, model-guided approaches are hampered by low enzymes'  sequence identity. CYPs from all kingdoms of life share six distinct protein areas, or domains, known as Substrate Recognition Sites (SRS1-6). The SRS regions are part of the CYP active site surrounding the heme prosthetic group [13] and contribute to the catalytic activity, substrate recognition or substrate binding specificity. The SRS regions can be effectively identified and targeted for mutagenesis by comparing the amino acid sequences of the *C. forskohlii* CYPs with previously characterized enzymes [14–16].

Here we report the optimization of the activity and specificity of CYP76AH15, which catalyzes the conversion of 13*R*-manoyl oxide to 11-oxo-13*R*-manoyl oxide [5]. To achieve this, a semi-rational site-directed mutagenesis approach targeting the six SRS domains was used. The activities of the obtained mutant variants were monitored by expressing them  in yeast cells producing 13*R*-manoyl oxide or miltiradiene, and  by study the in vivo diterpenoid accumulation.

## Results

### Defining the SRS regions of the *C. forskohlii* CYP76AH enzymes

**Fig. 2:**
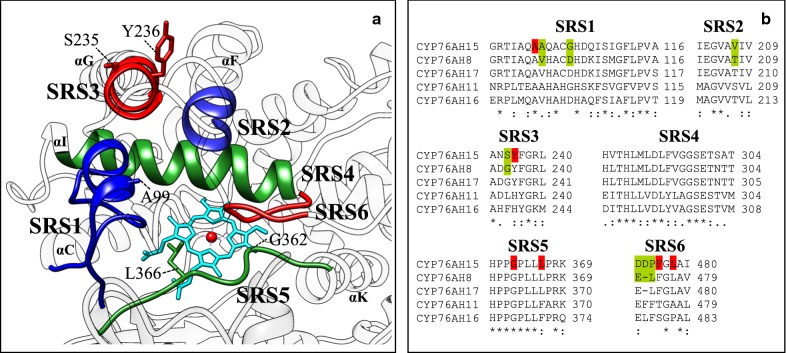
The SRS regions of selected CYP76AHs from *Coleus forskohlii.* **a** Homology model of CYP76AH15 with visualized SRS regions and selected helix-letters. SRS1, blue (left); SRS2, blue (right); SRS3, red (top); SRS5, green (bottom); and SRS6, red (right) were chosen as targeted areas for mutagenesis. The position of SRS4 on αI is shown in green. **b** Sequence alignments of the defined SRS regions of selected CYP76AH enzymes. Sites for mutagenesis are highlighted: Red; previously identified sites in other CYPs shown to confer changes in CYP properties (e.g. the SRS5 residues) or residues chosen in this work due to their structural positions. Green; sites of reciprocal mutagenesis of CYP76AH15 in comparison to CYP76AH8

The putative substrate recognition sites (SRS) of the *C. forskohlii* CYP76AH enzymes were identified by alignments and comparisons of reported SRSs of *Rattus norvegicus* CYP2A1 [17], *Hyoscyamus muticus* CYP71D55 [14] and *Thapsia villosa* CYP71AJ6 [18] (Additional file 1: Fig. S1). Furthermore, comparative homology modeling was used to determine and visualize the SRS regions of CYP76AH15 (Fig. 2a) to verify that the identified regions corresponded to the structural elements associated with the respective SRSs. According to this data, CYP76AH11, CYP76AH15 and CYP76AH16 contain 78 residues in the identified SRS regions whereas CYP76AH8 and CYP76AH17 contain 77 residues due to a deletion of one amino acid in the SRS6 (Fig. 2b).

The mutagenesis efforts we implemented aimed at increasing the efficiency of the first putative step of the forskolin pathway, namely the conversion of 13*R*-manoyl oxide to 11-oxo-13*R*-manoyl oxide (Fig. 1). Three CYP76AH enzymes from *C. forskohlii*—CYP76AH8, CYP76AH15 and CYP76AH17—were previously found to use 13*R*-manoyl oxide as substrate for the synthesis of 11-oxo-13*R*-manoyl oxide (Fig. 1), which is the main product of these enzymes, while other related diterpenoids were observed as minor compounds. CYP76AH15 was however shown to be more efficient towards the synthesis of 11-oxo-13*R*-manoyl oxide in comparison to its counterparts [5]. Because of the functional similarities observed between these enzymes, their SRS regions were compared to identify possible similarities and differences in their primary structure (Fig. 2b). CYP76AH8 and CYP76AH17 share similar product patterns when 13*R*-manoyl oxide is used as substrate and this is reflected also by their sequence identity which is 88%, when the full length sequences are compared. The sequence identity between CYP76AH8 and CYP76AH17 in the SRS regions is 99%, with a single amino acid difference in SRS1 (A117S, in CYP76AH17), demonstrating high sequence conservation, specifically in the SRS regions. Differences in the SRS regions between CYP76AH15 and CYP76AH8/CYP76AH17 were positioned mainly in SRS1, SRS3 and SRS6, whereas the SRS5 was conserved between all three enzymes (Table 1).

**Table 1:** Sequence identity comparisons of the SRS regions of selected CYP76AHs as well as of the full-length protein sequences

Aligned	SRS1 (%)	SRS2 (%)	SRS3 (%)	SRS4 (%)	SRS5 (%)	SRS6 (%)	Total SRS (%)	Total sequence (%)
AH15 and AH8	79	88	75	89	100	50	82	87
AH15 and AH17	75	88	75	89	100	50	81	80
AH8 and AH17	96	100	100	100	100	100	99	88
AH11 and AH16	54	88	50	89	82	63	71	76
AH15 and AH11	46	25	50	42	82	25	46	51
AH15 and AH16	50	25	25	47	82	25	46	54

CYP76AH11 and CYP76AH16, the other CYP76AHs involved in forskolin biosynthesis, catalyze distinct reactions in the pathway. CYP76AH11 is a multifunctional enzyme mainly catalyzing three oxygenations, and CYP76AH16 is regio-specific towards C-9 hydroxylation [5]. The SRS differences between CYP76AH11 and CYP76AH16 shared a similar pattern as when CYP76AH8/17 are compared with CYP76AH15. The major differences were found in SRS1 (54% identity), SRS3 (50% identity) and SRS6 (63% identity). SRS5 was not conserved between CYP76AH11 and CYP76AH16 (Table 1).

### Reciprocal and targeted mutations of CYP76AH15

We have focused our mutagenesis efforts on areas in the SRS regions that could be responsible for the observed catalytic differences, and thus responsible for the product specificity. When compared to the CYP76AH enzymes, the highest sequence variations were observed between the SRS1, SRS3, and SRS6 regions. This finding led us to target these areas for engineering. The SRS5 region was also included as it had been  previously identified as a mutagenesis hot spot, especially the residues located 5 and 9 amino acids upstream of the conserved ExxR motif [19]. Additionally, we targeted specific residues of the CYP76AH15 that corresponded to structurally equivalent positions with ones that have been found to increase activity or specificity, when subjected to mutagenesis in other plant CYPs. We furthermore used reciprocal mutagenesis with residues from CYP76AH8 to gain insights into the catalytic differences between the two enzymes (CYP76AH8 versus CYP76AH15).

Residue A99 was targeted as it was found to be structurally equivalent to position L123 in CYP720B1 (Additional file 1: Fig. S2), which was shown to increase diterpene turnover in yeast, when subjected to mutagenesis [20]. Mutations of Y236 (in SRS3) alone and in combination with S235 were chosen as both residues are situated at the N-terminal of the αG helix pointing outwards, according to the homology model (Fig. 2a), in an area which is suggested to be close to the ER membrane, in membrane-anchored CYPs [21, 22]. Residues G362 (5 residues from ExxR) and L366 (9 residues from ExxR) from SRS5 domain, were chosen for mutagenesis as both pointing towards the heme moiety of CYP76AH15 (Fig. 2). Mutations of structurally equivalent positions had previously yielded plant CYPs variants with altered catalytic characteristics [14–16, 20]. The targeted SRS6 residues were chosen, because according to previous studies, mutagenesis in this area had been found effective [23].

### CYP76AH15 SRS variants showing increased levels of 11-oxo-13R-manoyl oxide

Mutants were generated using the USER system as described in the Methods section. The generated CYP76AH15 variants were integrated into the genome of the previously reported EYS4498 yeast strain producing 13*R*-manoyl oxide (**1**), together with the cytochrome P450 oxidoreductase (POR) from *C. forskohlii, Cf*POR (Table 2). Our initial screening efforts were conducted with yeast cells growing in microtiter plates, allowing easy handling and screening of high numbers of CYP76AH15 mutant variants.

**Table 2:** Yeast strains utilized and generated in this study

Strain	Genotype	Source
S288C	MATα, SUC2, gal2, mal2, mel, flo1, flo8-1, ho, bio1, bio6, ura3Δ	Evolva A/S
EFSC4498	S288C, XI-2::(pPGK1-*Cf*TPS3/pTEF1-*Cf*TPS2/pTPI1-SpGGPPS7), ura3Δ	Evolva A/S
MO (−)	YFSC4498, XI-5::(pTEF2-*Cf*POR/URA3)	This work
MO-AH15	YFSC4498, XI-5::(pTDH3-CYP76AH15/pTEF2-*Cf*POR/URA3)	This work
MILT	S288C, XI-2::(pPGK1-*Cf*TPS3/pTEF2-*Cf*TPS3/pTPI1-SpGGPPS7), ura3Δ	This work
MILT (−)	MILT, XII-5::(pTEF2-*Cf*POR/URA3)	This work
MILT-AH15	MILT, XI-5::(pTDH3-CYP76AH15/pTEF2-*Cf*POR/URA3)	This work
FORSK	EFSC4498, X-3::(pTEF2-*Cf*POR/pFBA1-CYP76AH15/pSED1-CYP76AH11/pTDH3-CYP76AH16/pTEF2-*Cf*ACT1-8/URA3)	This work

**Fig. 3:**
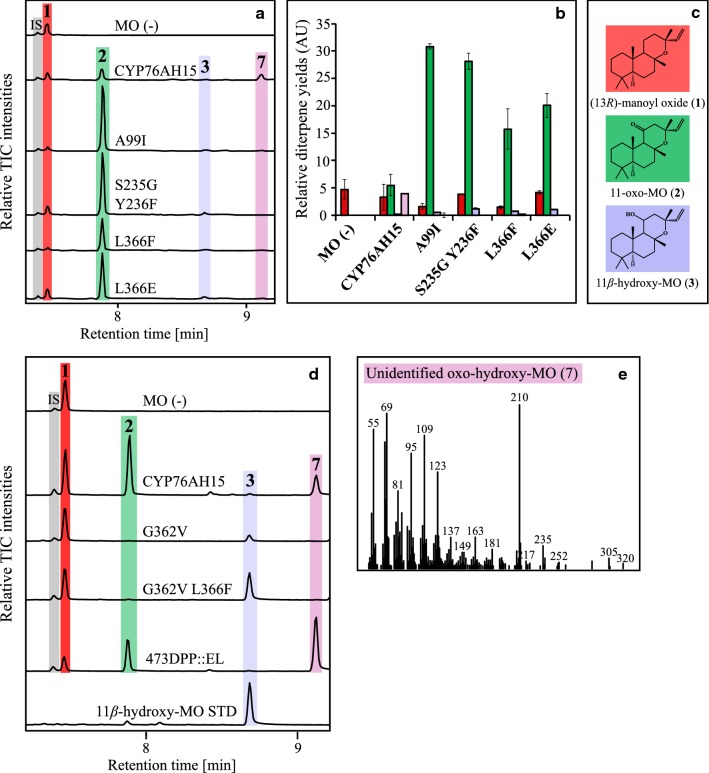
Diterpenoid product profiles from the CYP76AH15 mutant variants, in comparison to the native enzyme when expressed in yeast cells producing 13*R*-manoyl oxide (MO). **a** GC–MS chromatograms of the control strain MO (−) with *Cf*POR only, and strains from CYP76AH15 mutant-variants at the SRS1, SRS3 and SRS5 regions showing enhanced production of 11-oxo-13*R*-manoyl oxide (**2**). **b** Relative yields of the diterpenes **1** (red bars), **2** (green bars), **3** (light purple bars) and unknown **7** (light pink bars). The yeast strains expressing the SRS1, SRS3 and SRS5 variants of CYP76AH15 produced significantly higher levels of **2** and **3** and lower levels of **7**, in comparison to the native enzyme. **c** Chemical structures of the identified products formed by yeast. **d** GC–MS chromatograms of the control strain MO (−) with *Cf*POR only, native CYP76AH15, SRS5 and SRS6 mutated variants, and 11*β*-hydroxy-MO as internal standard (STD). **e** MS spectrum of (**7**) with parent ion *m*/*z* 320. *IS* internal standard (1 mg/L 1-eicosene), *MO* 13*R*-manoyl oxide, *TIC* total ion chromatogram. Error bars indicate standard deviation from three biological replicates

The control yeast strain (MO (−), harboring genome integrated *Cf*POR, no CYPs) was shown to accumulate 13*R*-manoyl oxide (**1**), when hexane extracts from yeast cells and broth were analyzed by GC–MS (Fig. 3a). The diterpenoids produced from strains harboring genome integrated native CYP76AH15 or mutant variants (Table 3, Additional file 1: Table S1) were analysed by GC–MS. Expression of CYP76AH15 led to the formation of 11-oxo-13*R*-manoyl oxide (**2**), 11*β*-hydroxy-13*R*-manoyl oxide (**3**) and an unknown compound (**7**) with a *m*/*z* 320 (Fig. 3e), which according to its molecular mass, could be an oxo-hydroxy-13*R*-manoyl oxide compound. Several CYP76AH15 mutant variants resulted in significant increase of 11-oxo-13*R*-manoyl oxide (**2**) (Fig. 3). More precisely, these variants were the A99I (SRS1), S235G-Y236F (SRS3), L366F and L366E (both in SRS5). Variant A99I displayed a 5.6-fold increase in accumulated 11-oxo-13*R*-manoyl oxide (**2**) while the levels of 13*R*-manoyl oxide (**1**) dropped significantly (P < 0.05, T-test) compared to the native CYP76AH15 (Fig. 3b). The variant A99I displayed no detectable levels (or trace amounts) of compound **7**, suggesting that this change resulted in an enzyme with a more specific activity towards the formation of 11-oxo-13*R*-manoyl oxide (**2**), although amounts of 13*R*-manoyl oxide (**1**) as well as of compound 11*β*-hydroxy-13*R*-manoyl oxide (**3**) could still be detected (Fig. 3b).

**Table 3:** Production levels of 11-oxo-13*R*-manoyl oxide (**2**) and 11-hydroxy-13*R*-manoyl oxide (**3**) by CYP76AH15 variants with SRS mutations using microtiter plates for yeast growth

Variant	SRS site(s)	2^a^	3^a^
Native CYP76AH15	–	1	1
A99I	1	5.6	3.0
S235G Y236F	3	5.1	7.3
L366F	5	2.9	4.2
L366E	5	3.7	6.2
A99I S235G Y236F	1 + 3	6.5	14.2
A99I L366F	1 + 5	6.2	4.9
S235G Y236F L366E	3 + 5	3.1	16.5
A99I S235G Y236F L366F	1 + 3+5	3.2	31.4

^a^Fold change of products **2** and **3**, respectively, compared to native CYP76AH15

### CYP76AH15 variants with altered product patterns

Among the generated CYP76AH15 variants with mutations at the SRS5 and SRS6 (Table 1, Additional file 1: Table S1), three variants were found to produce considerably different product patterns when compared to the native CYP76AH15. Two of them were within the SRS5, namely G362V and G362V L366F and one in SRS6, the deletion 473DDP::EL (Fig. 3d). Variant G362V produced small amounts of 11*β*-hydroxy-13*R*-manoyl oxide (**3**), while the double variant G362V L366F produced mainly 11*β*-hydroxy-13*R*-manoyl oxide (**3**) with trace amounts of 11-oxo-13*R*-manoyl oxide (**2**). The 473DPP::EL variant displayed a change in product pattern, as it produced significantly (P < 0.05, T-test) higher levels of the unknown compound **7**, while maintaining similar levels of 11-oxo-13*R*-manoyl oxide (**2**) when compared to the native enzyme (Fig. 3d).

### CYP76AH15 combinatorial mutagenesis with synergistic and antagonistic effects

After demonstrating that the change of a single residue in CYP76AH15 can cause a substantial effect in the enzyme’s product profile, we next sought to study the effect of combinatorial mutagenesis using the variants already tried from SRS1, SRS3 and SRS5 (Table 3), which resulted in increased formation of 11-oxo-13*R*-manoyl oxide (**2**). Combination of the mutations A99I, S235G and Y236F (SRS1 + SRS3) led to a variant able to accumulate 6.5 times more 11-oxo-13*R*-manoyl oxide (**2**) in comparison to the native CYP76AH15, indicating a synergistic effect of these mutations. Similarly, when the mutations A99I and L366F (SRS1 + SRS5) were combined, an increase of 6.2-fold was observed. The combination of A99I and L366E (SRS1 + SRS5) mutations resulted in an inactive enzyme. Combination of the mutations S235G, Y236F and L366E (SRS3 + SRS5) resulted in a 3.1-fold increase in the accumulation of 11-oxo-13*R*-manoyl oxide (**2**) in comparison to the 3.7-fold increase obtained with the L366E variant alone, indicating a slightly antagonistic effect. The combination of mutations A99I, S235G, Y236F and L366F (SRS1 + SRS3 + SRS5) offered a 3.2-fold increase in the production of 11-oxo-13*R*-manoyl oxide (**2**), again demonstrating a slightly antagonistic effect of the combined mutations. The combined mutations of A99I S235G Y236F L366E resulted in an inactive enzyme. A summary of the results from the screening of the mutant variants is shown at Additional file 1: Table S1. The combinatorial variants were also studied for their capacity to produce 11*β*-hydroxy-13*R*-manoyl oxide (**3**) as it is also a product of the native CYP76AH15. It was shown that besides affecting the formation of 11-oxo-13*R*-manoyl oxide (**2**) some of these variants resulted in a dramatic increase in the formation of 11*β*-hydroxy-13*R*-manoyl oxide (**3**). Variant A99I S235G Y236F L366F for example showed 31.4-fold increase regarding 11*β*-hydroxy-13*R*-manoyl oxide (**3**), indicating that the ability of this enzyme to actively catalyze the formation of ketone group in carbon 11 has been slightly shifted. Interestingly, the variant A99I contained the lowest change regarding accumulation of 11*β*-hydroxy-13*R*-manoyl oxide (**3**) and one of the highest increase observed regarding the accumulation of 11-oxo-13*R*-manoyl oxide (**2**) (Table 3).

### CYP76AH15 SRS6 variants towards miltiradiene conversion

CYP76AH15 was previously shown to be multifunctional as it not only produces 11-oxo-13*R*-manoyl oxide (**2**) from 13*R*-manoyl oxide (**1**) but also ferruginol, when miltiradiene is supplied as substrate [5]. We therefore investigated the effects of the generated CYP76AH15 mutants (Additional file 1: Table S1) regarding their catalytic activity towards ferruginol, in the presence of miltiradiene.

**Fig. 4:**
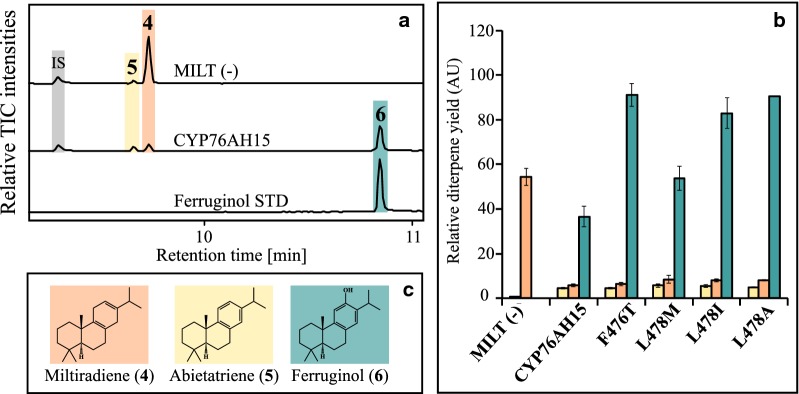
Effect of CYP76AH15 mutants on the synthesis of ferruginol in miltiradiene producing yeast strains. **a** GC–MS chromatograms of control MILT(−) strains expressing *Cf*TPS1, *Cf*TPS3 and *Cf*POR able to produce miltiradiene (**4**) and abietatriene (**5**). Expression of native CYP76AH15 lead to production of **6** which corresponds to ferruginol according to its comparison with an authentic standard (STD), retention time and fragmentation pattern. **b** Enhanced production of **6** from CYP76AH15 mutants, altered in the SRS6 region. **c** Chemical structures of the identified products produced by the yeast strains mentioned in **a**

A yeast strain producing miltiradiene (**4**) was constructed to explore first if CYP76AH15 facilitates the formation of ferruginol (**6**) in yeast, as it had previously been observed by transient expression in tobacco and if the generated mutations would affect ferruginol formation. The control yeast strain, {MILT (−)}, expressing *Cf*POR and no CYPs, was able to produce two compounds, miltiradiene (**4**) and abietatriene (**5**) (Fig. 4a). Expression of the native CYP76AH15 together with *Cf*POR led to formation of compound **6,** which matched the retention time and spectrum of ferruginol (Fig. 4a). From the mutants expressed in MILT (−) strain, only the SRS6 variants F476T and L478M/I/A showed increased production of ferruginol (**6**), with the highest ones observed from F476T and L478A giving a 2.4-fold increase (Fig. 4b). Interestingly, these variants did not affect the formation of 11-oxo-13*R*-manoyl oxide (**2**). The variants A99I, S235G, Y236F, L366F and L366E that showed improved formation of 11-oxo-13*R*-manoyl oxide (**2**) when supplied with 13*R*-manoyl oxide, showed lowered production of ferruginol (**6**) (Additional file 1: Table S1), indicating that these specific changes were specifically affecting the formation of 11-oxo-13*R*-manoyl oxide (**2**) but not of ferruginol (**6**).

### Improved CYPs efficiency in yeast strains growing in shake-flasks

**Fig. 5:**
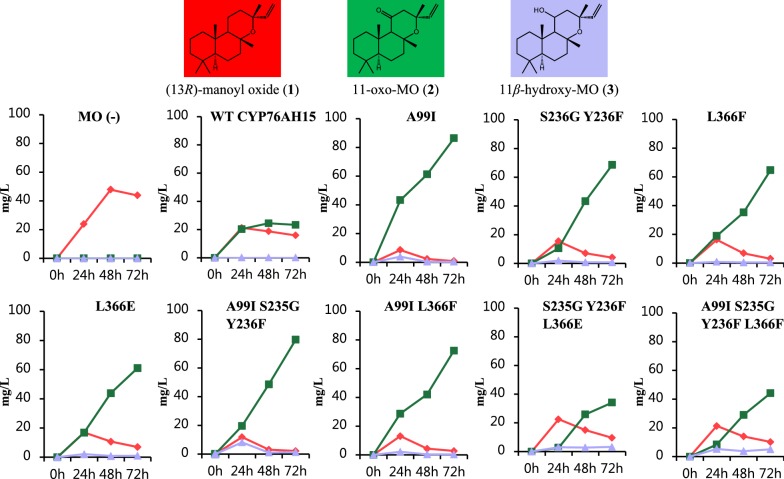
Production titers of **1** (red line), **2** (green line), **3** (purple line) and non-identified oxo-hydroxy-13*R*-manoyl oxide (**7**) (pink line, WT CYP76AH15 only) produced by engineered yeast strains expressing *Cf*TPS2, *Cf*TPS3, *Cf*POR and either wild type or selected mutant-variants of CYP76AH15, grown in shake-flasks

When comparing the remaining 13*R*-manoyl oxide (**1**) in strains expressing the native CYP76AH15 and the mutants, the levels of (**1**) were similar, despite the differences observed at the individual CYP products, when yeast strains were grown in microtiter plates. Since the yeast growth environment could affect the efficiency of CYPs, we explored if changes in growth conditions could improve CYPs’ performances. Thus, we utilized shake-flasks instead of microtiter plates as they offer improved oxygenation conditions. Yeast strains expressing selected CYP76AH15 variants, including the native enzyme, were grown in shake-flasks while in vivo production of diterpenoids was monitored over 72 h (Fig. 5, Table 4).

**Table 4:** Production titers of 13*R*-manoyl oxide (**1**), 11-oxo-13*R*-manoyl oxide (**2**) and 11-hydroxy-13*R*-manoyl oxide (**3**) by CYP76AH15 variants with SRS mutations using shake-flasks for yeast growth

Name	Total mg/L	Oxygenated (%)	1 mg/L	2 mg/L	3 mg/L	Content of 2 (%)
MO (−)	43.8	0	43.8	0.0	0.0	0
WT CYP76AH15	43.8	64	15.9	23.4	0.2	53
A99I	87.7	99	0.9	86.4	0.4	99
S235G Y236F	73.5	94	4.1	68.6	0.8	93
L366F	68.2	96	3.1	64.7	0.5	95
L366E	69.2	90	7.0	61.2	1.0	88
A99I S235G Y236F	83.4	97	2.2	79.8	1.4	96
A99I L366F	75.8	96	2.8	72.6	0.4	96
S235G Y236F L366E	47.1	80	9.6	34.2	3.3	73
A99I S235G Y236F L366F	59.6	83	10.2	44.2	5.1	74

Production of 13*R*-manoyl oxide (**1**) in the MO (−) control yeast strain stagnated after 48 h at approximately 43 mg/L, with a final titer of 43.8 mg/L after 72 h. The product of the native CYP76AH15 followed a similar pattern with stagnation in diterpene production after 48 h and a final total diterpene production of 43.8 mg/L while only a 64% fraction of diterpenoids was oxygenated (compounds **2**, **3** and **7**). The production of 11-oxo-13*R*-manoyl oxide (**2**) followed a similar stagnation pattern and reached 23.4 mg/L after 72 h, accounting for 53% of the total diterpene content. Introduction of selected variants resulted in a different production pattern with no stagnation regarding the accumulation of 11-oxo-13*R*-manoyl oxide (**2**), for all variants tested. The A99I variant showed a two-fold increase in total amounts of diterpenes (87.7 mg/L) compared to the native CYP76AH15 (43.8 mg/L) of which production of 11-oxo-13*R*-manoyl oxide (**2**) was 3.7-fold higher and accounting for 99% (86.4 mg/L) of the total diterpenoids. Levels of 13*R*-manoyl oxide (**1**) were nearly depleted with 0.9 mg/L remaining, a value 18-fold lower than when the native CYP76AH15 was used.

An increased accumulation of 11-oxo-13*R*-manoyl oxide (**2**) was observed from all the variants tested and accounted for > 93% of the total diterpene content for five of them (A99I, S235G Y236F, L366F, A99I S235G Y236F and A99I L366F). The titers were at least 3 times higher and reaching > 70 mg/L in three variants (A99I, S235G L236F and A99I S235G Y236F). The relative  increase observed regarding the accumulation of 11-oxo-13*R*-manoyl oxide (**2**) was generally lower in shake-flasks compared to microtiter experiments e.g. with the A99I S235G Y236F variant affording 6.5-fold increased levels in microtiter plates while a 3.1-fold increase in shake-flasks, compared with native CYP76AH15.

### Production of forskolin in yeast strains expressing native CYP76AH15 or A99I

**Fig. 6:**
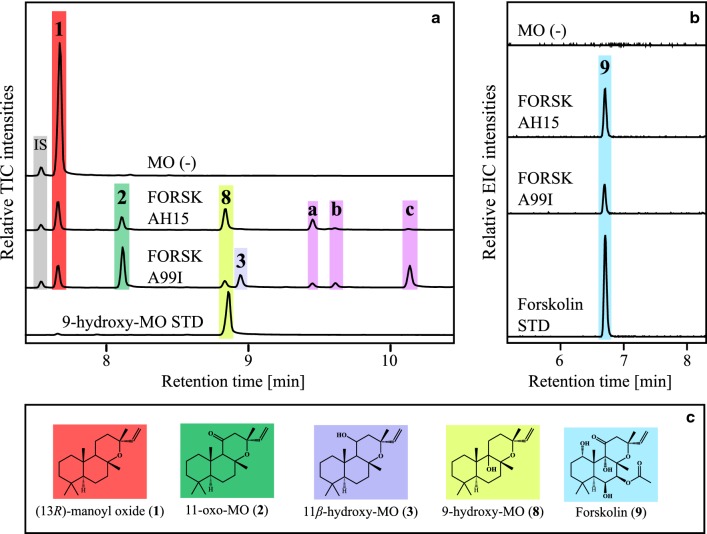
Production of forskolin and intermediates in yeast strains expressing native CYP76AH15 or the A99I  mutant  variant. **a** GC–MS chromatograms of the control strain MO (−) expressing *Cf*POR only, FORSK AH15 and FORSK A99I yeast strains expressing either native CYP76AH15 or A99I, respectively, together with the rest of the forskolin biosynthetic genes (CYP76AH11, CYP76AH16, *Cf*ACT1-8 and *Cf*POR). FORSK A99I showed increased accumulation of **2**, **3** and the unidentified intermediates **b** and **c**, whereas production of **1**, **8** and **a** was lowered compared to FORSK AH15. **b** Relative extracted ion chromatograms corresponding to [M+H]^+^ of forskolin at m/z 411.2381 ± 0.05 by LC–MS analysis. The analysis showed lowered production of forskolin in the strain FORSK A99I compared to FORSK AH15. **c** Identified products on GC–MS and LC–MS. *IS* internal standard (10 mg/L 1-eicosene), *MO* 13*R*-manoyl oxide, *TIC* total ion chromatogram, *EIC* extracted ion chromatograms

We next sought to explore the effect that engineered CYP76AH15 variants could have on forskolin production. For the trials, the A99I mutant variant was selected as it showed the highest levels of 11-oxo-13*R*-manoyl oxide (**2**) while maintaining low levels of 13*R*-manoyl oxide (**1**), 11*β*-hydroxy-13*R*-manoyl oxide (**3**) and **7**. The forskolin biosynthetic genes, CYP76AH11, CYP76AH15, CYP76AH16, *Cf*ACT1-8 and *Cf*POR were integrated into the genome of the strain EYS4498, in the X-3 locus. The generated strains were grown in baffled shake-flasks to maximize oxygen availability. Diterpene production in vivo was monitored after 72 h by both GC–MS and LC–MS (Fig. 6, Table 5).

**Table 5:** Relative diterpene yields in forskolin strains

Strain	1	2	3	8	9	a	b	c
MO (−)	4.1	nd	nd	nd	nd	nd	nd	nd
FORSK AH15	1	1	nd	1	1	1	1	1
FORSK A99I	0.5	2.1	–	0.2	0.5	0.3	3.3	22.7

*nd* not detected

Strains expressing the full forskolin pathway, with the native CYP76AH15 (FORSK AH15) or the CYP76AH15 A99I variant (FORSK A99I) showed production of **1**, **2**, **3**, 9-hydroxy-13*R*-manoyl oxide (**8**), forskolin (**9**) as well as several putative unidentified forskolin-intermediates (**a**, **b** and **c**). Yeast cells harboring the A99I mutant showed 2.1-fold increased production of **2** and decreased levels of **1** (Table 5), indicating a higher turnover of 13*R*-manoyl oxide by the A99I mutant compared to the native enzyme. The peaks of compounds **a**, **b** and **c** varied between FORSK AH15 and FORSK A99I (Table 5). Intermediate **a** was lower in FORSK A99I strain, whereas the intermediates **b** and **c** were 3.3 and 22.7-fold higher, respectively, compared to strain FORSK AH15. Forskolin production (**9**) was however 50% lower in strains expressing A99I compared to the native CYP76AH15 sequence. FORSK AH15 strains produced a final titer of 1.3 mg/L (± 0.12 mg/L) of forskolin whereas FORSK A99I produced 0.7 mg/L (± 0.01 mg/L) of forskolin.

## Discussion

Synthesis of the diterpene hydrocarbon backbones found in nature, such as 13*R*-manoyl oxide, is catalyzed by diterpene synthases. Production of these diterpenoid backbones can be achieved by heterologous expression of the relevant diterpene synthases in engineered yeast affording titers of more than 300 mg/L [9, 24, 25]. The structurally diverse backbones are then subjected to regio-selective functionalization by oxygenation reactions catalyzed by CYPs. CYPs are heme-containing enzymes found in all biological kingdoms. The primary sequences of CYPs are highly variable but their core protein fold is conserved from microbes to plants and consists of five β-sheets (named 1–5) and 13 α-helices (named αA–αL). SRS (substrate recognition sites) regions that span all CYP peptides have been assigned as important domains for substrate recognition and catalytic activity [13, 17]. In contrast to the diterpene synthases, CYPs are membrane bound enzymes, thus their  efficient expression in heterologous microbial systems is often challenging [4, 7, 10] and usually requires careful and complex optimization [26]. In bioengineering projects, CYP catalysed steps are likely to be rate-limiting. Additional challenges are imposed when it comes to the expression of multi-steps pathways, often consisting of more than one promiscuous CYP, as reported for the carnosic acid pathway containing two CYPs [7] or for the forskolin pathway containing three CYP enzymes [5]. Due to the important and unique catalytic activities of CYPs, several strategies have been adopted to improve or alter their performance [27–29]. It is believed that the ability of CYPs to tolerate engineering and still maintain their catalytic activities, originates from the non-polar residues in their active sites. The weak van der Waals force-interactions between the non-polar residues found at the catalytic pockets of these enzymes facilitate this phenomenon. In contrast, if stronger forces, such as hydrogen bonds or salt bridges, are altered between charged amino acids, this could easily negatively affect the protein stability and overall folding of enzymes [28].

Mutagenesis studies on membrane bound plant CYPs have been largely carried out in vitro and the functional changes observed mainly affected regio-specificity [15, 16], substrate specificity and catalytic efficiency [14]. In the present study, we have chosen to perform our experiments in vivo, because of the large differences observed between results obtained from in vivo versus in vitro experiments, as for example for the diterpene taxadiene-5*α*-hydroxylase in yeast [30]. In plant terpenoid metabolism, engineering of CYP720B1 (PtAO) and heterologous expression of the variants in yeast, resulted in increased accumulation of the enzyme’s product as well as in changes in substrate specificity and product profiles [31]. However, to utilize directed mutagenesis as a standardized tool for CYP optimization and directed outcomes, more precise knowledge is needed regarding regions and residues that could affect the functional properties of the enzyme.

Mutation strategies based on directed-evolution by error-prone PCR or DNA shuffling require a labor intensive screening of large libraries of CYP variants [27]. In the work described here, semi-rational engineering of SRS regions of CYP76AH15 from *C. forskohlii* was carried out to identify specific residues that dictate product specificity and enzyme efficiency, with the ultimate experimental goal to increase in vivo formation of the forskolin precursor 11-oxo-13*R*-manoyl oxide (**2**) in yeast. The activity of the generated CYP76AH15 variants was monitored by expressing them in yeast cells, engineered to produce the enzyme’s substrate, 13*R*-manoyl oxide, and identify and quantify the produced diterpenoids. In previous targeted studies on mutagenesis of plant CYPs, residues have been chosen according to their position in relation to heme, i.e. residues defining the cavity enclosing the heme-containing catalytic pocket of the enzyme [14, 16, 32]. This approach reduces the number of residues targeted for mutation making it easier to accomplish, but it has the risk to miss residues with profound effect on the catalytic activity. In our current study, homology modelling demonstrates that the A99I mutation in SRS1 and the mutations S235G Y236F in SRS3 are positioned ~ 18Å and ~ 25Å away from the heme–iron atom, respectively (Fig. 2a). Moreover, the S235 and Y236 side chains are pointing outwards away from the heme, according to our homology models. The A99I and S235G Y236F mutations gave the most profound increase in 11-oxo-13*R*-manoyl oxide (**2**) production. These residues may play an important role in the orientation of 13*R*-manoyl oxide (**1**) in the enzymatic cavity and thus force exclusive oxygenation of carbon 11 leading to increased synthesis of 11-oxo-13*R*-manoyl oxide (**2**) (Fig. 3). These residues would not have been selected for mutagenesis based strictly on criteria regarding their position relative to heme. Other mutagenesis studies have been based on substitution of amino acids with other counterparts bearing similar properties [31], like the case of A99I mutation where both amino acids (A, alanine and I, isoleucine) are lipophilic. However, this is not the case with the L366E or S235G Y236F substitutions, where a lipophilic amino acid is replaced with an acidic (L366E), or polar amino acids  are substituted with lipophilic ones (S235G Y236F). Interestingly, the latter substitutions provided higher enzyme efficiency, but not necessarily specificity (Table 3).

In microtiter plate experiments, the A99I mutation in SRS1 was responsible for the highest increase in the production of 11-oxo-13*R*-manoyl oxide (**2**) and showed the highest product specificity (Fig. 3). In shake-flask experiments, the mutation enabled nearly full conversion of 13*R*-manoyl oxide (**1**) into 11-oxo-13*R*-manoyl oxide (**2**), (Fig. 5; Table 4). Residue A99 was targeted for mutagenesis because it is positioned in a structurally equivalent position to L123 in CYP720B1 (Fig. S2), which was previously shown to facilitate significant increase in production of oxygenated diterpenoids in yeast [31]. Both A99 in CYP76AH15 and L123 in CYP720B1 are positioned in the first turn of αB′ (Fig. 2) pointing inwards towards the heme cavity, suggesting that residues in this particular position are important for the catalytic properties of CYPs and could thus be effective targets for future mutagenesis studies. The ability of CYP76AH15 to synthesize ferruginol (**6**) was reduced by the A99I mutation (Additional file 1: Table S1), suggesting that the mutation altered product specificity by changing the enzymatic cavity and the way the enzyme interacts with its substrates.

In microtiter plates, the double mutation S235G Y236F in SRS3 afforded a 5.2-fold increase in the formation of 11-oxo-13*R*-manoyl oxide (**2**) (Table 3). In shake-flask conditions, the S235G Y236F mutation increased the oxygenation levels of 13*R*-manoyl oxide (**1**) with the majority being transformed into 11-oxo-13*R*-manoyl oxide (**2**), (93% content of **2**), (Table 4). The S235 and Y236 residues in SRS3 are positioned in the N-terminal of the αG (Fig. 2) and in the loop connecting αF′ and αG, a region that has been suggested to be directly interacting with the lipid bilayer [21, 22, 33]. The S235G Y236F mutations may facilitate the access of the substrate in the catalytic cavity through the membrane, because 13*R*-manoyl oxide is a highly lipophilic substrate and SRS3 together with SRS2 form the entrance channel towards the heme, through the lipid bilayer [13, 21, 22]. Mutagenesis of structurally equivalent positions in other plant CYPs has not been reported to our knowledge. It thus remains to be investigated whether SRS3 mutagenesis at these positions affects other plant CYPs using different substrates. The S235G Y236F variant retained the activity towards ferruginol (**6**) production (Additional file 1: Table S1) at levels similar to the native enzyme, suggesting that S235GY236F specifically affected conversion of 13*R*-manoyl oxide (**1**) into oxo-13*R*-manoyl oxide (**2**).

Specific changes in SRS5 led to an increase in the accumulation of either 11-oxo-13*R*-manoyl oxide (**2**) (L366F and L366E) or 11*β*-hydroxy-13*R*-manoyl oxide (**3**), (G362V and G362VL366F), the latter mutation resulting in an altered product profile. Thus, by changing residues in SRS5 it is possible to obtain CYP76AH15 variants with different functional properties ranging from specific producer of 11-oxo-13*R*-manoyl oxide (**2**) to a dedicated 11*β*-hydroxy-13*R*-manoyl oxide (**3**) synthase (Fig. 3). The G362V and L366E mutations are positioned five (G362) and nine (L366) residues from the conserved ExxR motif and are located in SRS5 positions known to be mutational “hotspots” [19]. Mutations in these positions have previously been shown to change the catalytic properties of plant CYPs [15, 16, 34]. Our study thus substantiates that these residues play a critical role in CYP functional properties.

Reciprocal mutations in the SRS6 region of the CYP76AH15 were guided by sequence-comparisons between the CYP76AH15 and CYP76AH8 enzymes. The 473DPP::EL variant showed increased formation of **7**, a product with a mass corresponding to oxo-hydroxy-13*R*-manoyl oxide (Fig. 3d). This result suggested that additions or deletions of residues represents an underexplored mutagenesis approach that could alter the catalytic properties of CYPs. Different mutations in the same region (like the F476T and L478 M/I/A) significantly increased the activity of CYP76AH15 towards the synthesis of ferruginol (**6**) when miltiradiene (**4**) is supplied as a substrate (Fig. 4). These mutations did not affect the activity of the enzyme when 13*R*-manoyl oxide (**1**) was used as substrate. This suggests that the altered amino acid residues at these positions may change the enzymatic cavity in a way that enables the additional docking of miltiradiene, as a substrate, without disruption of the binding site for 13*R*-manoyl oxide.

The generation of double or triple CYP76AH15 mutants by combining mutations in residues that have shown an increased enzymatic activity, resulted in synergistic as well as antagonistic effects and in one case even a catalytically impaired enzyme. The A99I S235G Y236F (SRS1 and SRS3) variant resulted in 6.5-fold higher amounts of 11-oxo-13*R*-manoyl oxide (**2**) and a simultaneous 14.2-fold increase of 11*β*-hydroxy-13*R*-manoyl oxide (**3**). A similar observation was made with the A99I L366F (SRS1 and SRS5) variant whereas the A99I L366E variant was inactive (Additional file 1: Table S1).

The relative  increase of 11-oxo-13*R*-manoyl oxide (**2**) production from CYP76AH15 mutants was observed to be lower when the yeast cells were growing in shake-flasks compared to microtiter plates. In parallel, lower levels of remaining 13*R*-manoyl oxide was observed in shake-flasks, indicating that the metabolic conversion of 13*R*-manoyl oxide (**1**) to 11-oxo-13*R*-manoyl oxide (**2**) was increased under these conditions when compared to microtiter plates. Increased oxygen levels in the flask cultures may explain this observation. This was especially obvious with the variants A99I, A99I S235G Y236F and A99I L366F that all showed at least fivefold increase in production of 11-oxo-13*R*-manoyl oxide (**2**) in microtiter plates whereas only threefold increase was obtained in shake-flasks. At the same time, the remaining 13*R*-manoyl oxide (**1**) in shake-flask cultures carrying strains with mutant-variants was more than five times less when compared with the residual levels of (**1**) from cultures with native CYP76AH15. For the A99I variant, particularly low residual amounts of 13*R*-manoyl oxide (< 1 mg/L) were observed, practically showing full turnover of 13*R*-manoyl oxide (**1**) to 11-oxo-13*R*-manoyl oxide (**2**). Strains harboring the optimized CYP76AH15 variants were therefore most likely substrate-limited under shake-flask conditions, which may have hindered higher final product titers. Previous studies have reported reduced yields of yeast-produced diterpenoids when moving from smaller to larger volume cultures mainly due to limited CYP activity, which is contrary to our findings [35].

Our analysis of the CYP76AH15 SRS variants reported here show that modifications of even a single amino acid residue can result in significant changes of the enzyme’s catalytic properties. The changes may be accompanied by a more narrow product profile such as exclusive formation of 11-oxo-13*R*-manoyl oxide (**2**) by the A99I variant or 11*β*-hydroxy-13*R*-manoyl oxide (**3**) by the G362VL366F variant (Fig. 3 and Additional file 1: Table S3). Interestingly, all the above functional variants were located in three out of the six SRS domains, namely SRS1, SRS3 and SRS5 (Table 3). Other studies have also reported that mutagenesis in SRS domains may lead to enzymes with improved or altered functional properties [13, 14, 31].

To study how the generated mutants can affect forskolin production, the entire forskolin biosynthetic pathway was reconstructed in yeast. Two different yeast strains producing forskolin were generated, the FORSK-AH15 (as control) which expresses the native CYP76AH15, together with all native forskolin biosynthetic enzymes, and the FORSK-A99I which expresses the mutant A99I instead of the native CYP76AH15. After 72 h of growth in shake-flasks, total diterpenoids were extracted from the two strains. It was shown that although the improved activity of mutant A99I towards synthesis of 11-oxo-13*R*-manoyl oxide (**2**), which is a pathway intermediate, the overall forskolin yield was lower by approximately 50% compared to the strain expressing the native CYP76AH15. We did, however, observe 2.1-fold higher accumulation of 11-oxo-13*R*-manoyl oxide (**2**) and lower accumulation of 13*R*-manoyl oxide (**1**), suggesting that the introduced A99I variant did indeed increase the turnover of the first step towards forskolin, which was our overall goal. In parallel our results show that the excess accumulation of compound **2** could not be used efficiently from the downstream enzymes (CYP76AH11 and CYP76AH16). The increased turnover of 13*R*-manoyl oxide (**1**) to 11-oxo-13*R*-manoyl oxide (**2**) did not result in higher flux towards the final production of forskolin, which could be caused by several factors: (i) the downstream performance of the remaining CYPs (CYP76AH11 and CYP76AH16) did not accommodate the increased accumulation of 11-oxo-13*R*-manoyl oxide, thus leading to a buildup of intermediates such as the unidentified compounds (**b**) and (**c**). The increased accumulation of 11-oxo-13*R*-manoyl oxide (**2**) might interfere with the activity of the downstream CYPs (feedback regulation or levels of accumulated 11-oxo-13*R*-manoyl oxide have reached the downstream enzymes saturation level). (ii) The native CYP76AH15 sequence has an unidentified role in the remaining hydroxylations of (**2**) towards forskolin. This role could be limited after the introduction of the A99I mutation. The native CYP76AH15 enzyme does indeed have the capability to hydroxylate (**2**) into compound (**7**), which might be a crucial intermediate towards forskolin and the substrate of one of the downstream CYPs. This could be investigated by incorporation of a native CYP76AH15 sequence in the FORSK A99I strain or the 473DPP::EL mutant that showed increased production of (**7**). At present, the exact order of hydroxylations towards forskolin remains unknown and because of the multifunctional nature of the involved CYPs, it is not clear which enzyme is responsible for each hydroxylation. For example, CYP76AH11 was been shown to be able to produce deacetylforskolin, by itself, nevertheless in very small amounts, when expressed in tobacco. It is possible that the multifunctionality observed in forskolin biosynthetic CYPs could be critical for maintaining the optimal flux through the pathway, for high forskolin synthesis. (iii) According to Laursen et al., [36], metabolic pathways, especially those that involve membrane bound enzymes like CYPs, can form metabolons (multi-enzymatic assemblies or complexes) which facilitate the pathway flux or product channeling, for optimizing pathway efficiency. It is possible that by changing the product profile of CYP76AH15 (and possibly other unknown properties, at the same time) we have interfered with the formation or stoichiometry of the forskolin metabolon, which in turn has disturbed the flux of the pathway. It is always challenging to reconstitute multi-enzymatic pathways and mimic the native conditions when using heterologous systems, different cell compartmentalization or heterologous gene regulatory elements (promoters). In cases like this, where we add an additional complexity to the system, like engineered enzymes, the balance and equilibrium of the pathway can be easily perturbed. This is an example that shows the very delicate design of biosynthetic pathways in nature and the difficult task of systems biology to unravel the complexity of plant biochemistry. In addition, one can speculate that the use of standardized parts and biobricks for metabolic engineering of host organisms, via synthetic biology strategies, may not be sufficient in all cases, or at least shows that much more data are needed for being able to design optimal biosynthetic hosts.

Plants may have evolved multifunctional CYP enzymes to lower the metabolic cost of specialized metabolite biosynthesis while retaining chemical diversity [37]. The interplay of CYP mutants with increased or altered functional properties may result in significant changes in the product profiles obtained. Our results show the plasticity of CYP enzymes, which is likely responsible for the remarkable diversity of this enzyme superfamily; this is reflected not only by the role of these enzymes in the metabolism of all kingdoms of life, but also by the vast number of family members that have resulted from gene duplications and neofunctionalization.

## Methods

### Sequences, alignments and SRS determination

Sequences utilized in this work are listed in Additional file 1: Table S2 together with primers in Additional file 1: Table S3. SRS regions in CYP76AH15 and CYP76AH8 were determined by aligning with previously established SRS regions in CYP2A1 from *Rattus norvegicus* (Genbank Accession number: P11711), CYP71D55 from *Hyoscyamus muticus* (Genbank Accession number: A6YIH8) and CYP71AJ6 from *Thapsia villosa* (Genbank Accession number: AKJ23347) [14, 17, 18] (Additional file 1: Fig. S1).

### Homology modeling

Homology modeling to manually inspect the putative SRS regions was carried out using UCSF Chimera version 1.10.2 (University of California) and Modeller 9.15 [38, 39]. BLAST searches in the PDB database was carried out using CYP76AH15 sequence as query to find templates (Table 6). Two templates were utilized for modeling using default settings in Modeller and inclusion of the HEME group from the 3RUK template.

**Table 6:** Templates used for homology modeling of CYP76AH15

Target	Templates	Sequence identity (%)	PDB number
CYP76AH15	CYP17A	29	3RUK_A
	CYP2A13	26	4EJI_A

### Site-directed mutagenesis and plasmid construction

Uracil-specific-excision-reagent (USER) cloning [40] was utilized for site-directed mutagenesis of CYP76AH15 and USER primers were designed using the online AMUSER tool [41]. Variants of CYP76AH15 were generated by fully amplifying cloning vector pJET1.2 (Thermo Fischer, USA) containing CYP76AH15 with USER specific site-directed mutagenesis primers and purification of PCR products using a PCR-purification kit (QIAquick PCR Purification Kit, Qiagen, USA), enzymatic treatment with DnpI (New England Biolabs, USA) and performing USER cloning and *Escherichia coli* transformation as previously described [41]. Plasmids were recovered using mini-prep kits (Qiaprep Spin Miniprep Kit, Qiagen, USA). CYP76AH15 variants from pJET1.2 were USER cloned into yeast single integration vectors and assembler vectors as previously described [9].

### *Saccharomyces cerevisiae* strain construction, growth and extraction of metabolites

Yeast strain EFCS4498 [5] was utilized as the parent strain for all generated yeast strains regarding 13*R*-manoyl oxide production and forskolin production (Table 3). All constructs were genomically integrated at the XI-5 or X-3 site [42]. Yeast strains producing miltiradiene were constructed as previously described [9] using the yeast assembler system and the XI-2 site [42] and strain S288C.

Yeast strains were transformed according to the lithium-acetate protocol [43]. Plasmids were digested with *Not*I (New England Biolabs, USA) to release integration cassettes and the digest mixes were added to the yeast transformation reactions without purification. Transformed yeast cells were selected on synthetic complete plates lacking uracil (SC-URA) prepared from drop-out powder (Sigma-Aldrich, Germany) and genotypes were confirmed by PCR.

Production of diterpenes in engineered *S. cerevisiae* was carried out as previously described in microtiter plate formats using feed-in-time (FIT) medium (m2p Labs, Germany) to mimick Fed-batch conditions [9]. Vitamins and enzyme solutions were added to final concentrations of 1% (v/v) and 0.8% (v/v), respectively in yeast strains producing 13*R*-manoyl oxide and miltiradiene. Pre-cultures were grown for 16 h (280 RPM at 30 °C) in 500 μL selective medium (SC-URA). Next, 50 μL of the pre-culture was transferred to 500 μL FIT medium in microtiter plates for diterpene production screening, over 72 h. Shake-flask experiments of CYP76AH15 mutants alone were carried out by innoculating 5 mL pre-cultures in selective medium (SC-URA) for 16 h (280 RPM at 30 °C) and transferring 3 mL pre-cultures to 25 mL FIT medium in 250 mL Erlenmeyer flasks that were left shaking for 72 h (180 RPM at 30 °C). Shake-flask experiments of strains producing forskolin were carried by inoculating 5 mL pre-cultures in selective medium (SC-URA) for 16 h (180 RPM at 30 °C) and transferring 1 mL pre-cultures to 9 mL FIT medium (0.5% enzyme mix added and 4% additional sugar added) in 100 mL baffled shake-flasks shaking for 72 h (180 RPM at 30 °C).

For gas-chromatography-mass-spectrometry (GC–MS) analysis, 0.4 mL of the yeast cells and broth was transferred to 1.5 mL glass vials and 0.4 mL *n*-hexane (GC grade) added, spiked with 1 mg/L 1-eicosene as internal standard. Samples were vortexed for 5 s and left shaking (> 200 RPM, room temperature, 1 h). Next, 200–300 μL of the hexane phase were transferred to new 1.5 mL glass vials with inserts ready for GC–MS analysis.

For liquid-chromatography-mass-spectrometry (LC–MS) analysis, 0.1 mL of the yeast cells and broth was transferred to 1.5 mL glass vials and added 0.4 mL acetonitrile (HPLC grade). Samples were vortexed 5 s and left shaking (> 200 RPM, room temperature, 1 h). Next, 200–300 μL were filtered and transferred to new 1.5 mL glass vials with inserts ready for LC–MS analysis.

### GC–MS analysis of diterpenes

A Shimadzu GCMS-QP2010 Ultra system fitted with an Agilent HP-5MS column (30 m × 0.25 mm i.d., 0.25 µm film thickness) was utilized for GC–MS analysis. Injection volume was set to 1 µL and the injection temperature at 250 °C with the following GC program for 13*R*-manoyl oxide related samples: 60 °C for 1 min, ramp at rate 30 °C/min to 180 °C, ramp at rate 10 °C/min to 280 °C and hold for 3 min. The total run time was 18 min. The temperature program for miltiradiene related samples was: 80 °C for 2 min, ramp at rate 30 °C/min to 170 °C and hold 3 min, ramp at rate 30 °C/min to 280 °C and hold for 3 min. The total run time was 14.67 min.

The ion source temperature of the mass spectrometer (MS) was set to 250 °C and spectra were recorded from 50 to m/z 350 (13*R*-manoyl oxide samples) and 50 to 400 m/z (miltiradiene samples). Compound identification was carried out using authentic standards and comparison to reference spectra in databases (Wiley Registry of Mass Spectral Data, 8th Edition, July 2006, John Wiley & Sons, ISBN: 978-0-470-04785-9) and previously reported spectra [4, 9]. Relative differences in diterpene yields were based on an average of the total ion chromatogram (TIC) peak area for the compound of interest normalized to the TIC area of the internal standard (IS). Compounds analyzed by GC–MS in this work were 13*R*-manoyl oxide (**1**), 11-oxo-13*R*-manoyl oxide (**2**), 11*β*-hydroxy-13*R*-manoyl oxide (**3**), miltiradiene (**4**), abietatriene (**5**), ferruginol (**6**), an unidentified oxo-hydroxy-13*R*-manoyl oxide compound (**7**) and 9-hydroxy-13*R*-manoyl oxide (**8**).

### Quantification of diterpenes produced by *Saccharomyces cerevisiae* by GC–MS

13*R*-manoyl oxide was obtained as previously described [44]. A standard of 11-oxo-13*R* manoyl oxide was isolated from 100 mL *S. cerevisiae* culture expressing SpGGPPS7, *Cf*TPS2, *Cf*TPS3, the A99I variant of CYP76AH15 and *Cf*POR, likewise using solid phase extraction (Dual Layer Florisil/Na_2_SO_4_ 6 ml PP SPE Tube, Sigma-Aldrich, USA) and eluted using a gradient of *n*-hexane containing EtOAc in concentrations of 0.25%, 0.5%, and 1–8%. 11-oxo-13*R*-manoyl oxide was obtained as 7 mg white powder upon evaporation of the 1–3% EtOAc containing fractions. The powder was resuspended in hexane and standard curves of both 13*R*-manoyl oxide and 11-oxo-13*R*-manoyl oxide were prepared spiked with 10 mg/L 1-eicosene as an internal standard. An unknown oxo-hydroxy-13*R*-manoyl oxide **7** was quantified assuming an identical response factor to 11-oxo-13*R*-manoyl oxide. Yeast cells and culture medium to be quantified for diterpene productions were extracted as previously described with *n*-hexane spiked with 10 mg/L 1-eicosene as an internal standard.

### LC–MS analysis of forskolin producing yeast strains

The Ultimate 3000 UHPLC + Focused system (Dionex Corporation, USA) was utilized as LC component while coupled to a Bruker Compact ESI-QTOF-MS (Bruker Daltonik, Germany) to analyze forskolin containing samples. Separation of samples was carried out using a Kinetex XB-C18 column (100 × 2.1 mm i.d., 1.7 μm particle size, 100 Å pore size; Phenomenex Inc., USA) maintained at 40 °C. The flow rate was set to 0.3 mL/min with a mobile phase consisting of 0.05% (v/v) formic acid in water (solvent A) and 0.05% (v/v) formic acid in acetonitrile (solvent B). A gradient LC method was used with solvent B at 20% for 30 s, ramped to 100% for 8.5 min, hold at 100% for 2 min and lowered to 20% over 30 s and final hold for 3.5 min. Total run time was 15 min. The ESI source parameters were as follows: capillary voltage, 4500 V, nebulizer pressure 1.2 bar; dry gas flow, 8 L/min and the dry gas temperature set to 250 °C. MS mode only was set on the qTOF-MS and the collision cell energy at 7 eV. Collision cell RF was set to 500 Vpp and ions were monitored in the positive mode over a range of 50–1300 *m/z* and spectra collected at a rate of 2 Hz.

## Additional file

**Additional file 1.** Additional tables and figures.

## Abbreviations

CYP: cytochrome P450
GGPP: geranylgeranyl diphosphate
CPP: copalyl diphosphate
8-OH-CPP: 8-hydroxy-copalyl diphosphate
SRS: substrate recognition site
diTPS: diterpene synthase
*Cf*POR: *Coleus forskohlii* cytochrome P450 oxidoreductase
*Cf*TPS1/2/3: *Coleus forskohlii* diterpene synthase 1/2/3
SC: synthetic complete
FIT: feed-in-time
MO: 13*R*-manoyl oxide

## References

[1] Godard MP, Johnson BA, Richmond SR, Michael P, Johnson BA, Richmond SR (2005). Body composition and hormonal adaptations associated with forskolin consumption in overweight and obese men. Obes Res.

[2] Seamon KB, Padgett W, Daly JW (1981). Forskolin: unique diterpene activator of adenylate cyclase in membranes and in intact cells. Proc Natl Acad Sci USA.

[3] Alasbahi RH, Melzig MF (2010). *Plectranthus barbatus*: a review of phytochemistry, ethnobotanical uses and pharmacology part 2. Planta Med.

[4] Ignea C, Ioannou E, Georgantea P, Trikka FA, Athanasakoglou A, Loupassaki S (2016). Production of the forskolin precursor 11β-hydroxy-manoyl oxide in yeast using surrogate enzymatic activities. Microb Cell Fact.

[5] Pateraki I, Andersen-Ranberg J, Jensen NB, Wubshet SG, Heskes AM, Forman V (2017). Total biosynthesis of the cyclic AMP booster forskolin from *Coleus forskohlii*. Elife.

[6] Pateraki I, Andersen-Ranberg J, Hamberger B, Heskes AM, Martens HJ, Zerbe P (2014). Manoyl oxide (13R), the biosynthetic precursor of forskolin, is synthesized in specialized root cork cells in *Coleus forskohlii*. Plant Physiol.

[7] Ignea C, Athanasakoglou A, Ioannou E, Georgantea P, Trikka FA, Loupassaki S (2016). Carnosic acid biosynthesis elucidated by a synthetic biology platform. Proc Natl Acad Sci.

[8] Guo J, Ma X, Cai Y, Ma Y, Zhan Z, Zhou YJ (2016). Cytochrome P450 promiscuity leads to a bifurcating biosynthetic pathway for tanshinones. New Phytol.

[9] Andersen-Ranberg J, Kongstad KT, Nielsen MT, Jensen NB, Pateraki I, Bach SS (2016). Expanding the landscape of diterpene structural diversity through stereochemically controlled combinatorial biosynthesis. Angew Chemie Int Ed.

[10] Guo J, Zhou YJ, Hillwig ML, Shen Y, Yang L, Wang Y (2013). CYP76AH1 catalyzes turnover of miltiradiene in tanshinones biosynthesis and enables heterologous production of ferruginol in yeasts. Proc Natl Acad Sci USA.

[11] Bak S, Beisson F, Bishop G, Hamberger B, Höfer R, Paquette S (2011). Cytochromes p450. Arabidopsis Book.

[12] Munro AW, Girvan HM, Mason AE, Dunford AJ, McLean KJ (2013). What makes a P450 tick?. Trends Biochem Sci.

[13] Gricman Ł, Vogel C, Pleiss J (2015). Identification of universal selectivity-determining positions in cytochrome P450 monooxygenases by systematic sequence-based literature mining. Proteins.

[14] Takahashi S, Yeo YS, Zhao Y, O’Maille PE, Greenhagen BT, Noel JP (2007). Functional characterization of premnaspirodiene oxygenase, a cytochrome P450 catalyzing regio- and stereo-specific hydroxylations of diverse sesquiterpene substrates. J Biol Chem.

[15] Schalk M, Croteau R (2000). A single amino acid substitution (F363I) converts the regiochemistry of the spearmint (−)-limonene hydroxylase from a C6- to a C3-hydroxylase. Proc Natl Acad Sci USA.

[16] El-Awaad I, Bocola M, Beuerle T, Liu B, Beerhues L (2016). Bifunctional CYP81AA proteins catalyse identical hydroxylations but alternative regioselective phenol couplings in plant xanthone biosynthesis. Nat Commun.

[17] Gotoh O (1992). Substrate recognition sites in cytochrome P450 family 2 (CYP2) proteins inferred from comparative analyses of amino acid and coding nucleotide sequences. J Biol Chem.

[18] Dueholm B, Krieger C, Drew D, Olry A, Kamo T, Taboureau O (2015). Evolution of substrate recognition sites (SRSs) in cytochromes P450 from Apiaceae exemplified by the CYP71AJ subfamily. BMC Evol Biol.

[19] Seifert A, Pleiss J (2009). Identification of selectivity-determining residues in cytochrome P450 monooxygenases: a systematic analysis of the substrate recognition site 5. Proteins Struct Funct Bioinform.

[20] Ignea C, Ioannou E, Georgantea P, Loupassaki S, Trikka FA, Kanellis AK (2015). Reconstructing the chemical diversity of labdane-type diterpene biosynthesis in yeast. Metab Eng..

[21] Jensen K, Osmani SA, Hamann T, Naur P, Møller BL (2011). Homology modeling of the three membrane proteins of the dhurrin metabolon: catalytic sites, membrane surface association and protein–protein interactions. Phytochemistry.

[22] Monk BC, Tomasiak TM, Keniya MV, Huschmann FU, Tyndall JD, O’Connell JD (2014). Architecture of a single membrane spanning cytochrome P450 suggests constraints that orient the catalytic domain relative to a bilayer. Proc Natl Acad Sci USA.

[23] Scheler U, Brandt W, Porzel A, Rothe K, Manzano D, Bozic D (2016). Elucidation of the biosynthesis of carnosic acid and its reconstitution in yeast. Nat Commun..

[24] Dai Z, Liu Y, Huang L, Zhang X (2012). Production of miltiradiene by metabolically engineered *Saccharomyces cerevisiae*. Biotechnol Bioeng.

[25] Trikka FA, Nikolaidis A, Athanasakoglou A, Andreadelli A, Ignea C, Kotta K (2015). Iterative carotenogenic screens identify combinations of yeast gene deletions that enhance sclareol production. Microb Cell Fact.

[26] Kitaoka N, Wu Y, Xu M, Peters RJ (2015). Optimization of recombinant expression enables discovery of novel cytochrome P450 activity in rice diterpenoid biosynthesis. Appl Microbiol Biotechnol.

[27] Behrendorff JBYH, Huang W, Gillam EMJ (2015). Directed evolution of cytochrome P450 enzymes for biocatalysis: exploiting the catalytic versatility of enzymes with relaxed substrate specificity. Biochem J..

[28] Jung ST, Lauchli R, Arnold FH (2011). Cytochrome P450: taming a wild type enzyme. Curr Opin Biotechnol.

[29] Janocha S, Schmitz D, Bernhardt R (2015). Terpene hydroxylation with microbial cytochrome p450 monooxygenases. Adv Biochem Eng Biotechnol.

[30] Edgar S, Zhou K, Qiao K, King JR, Simpson JH, Stephanopoulos G (2016). Mechanistic insights into taxadiene epoxidation by taxadiene-5α-hydroxylase. ACS Chem Biol.

[31] Ignea C, Ioannou E, Georgantea P, Loupassaki S, Trikka FA, Kanellis AK (2015). Reconstructing the chemical diversity of labdane-type diterpene biosynthesis in yeast. Metab Eng.

[32] Sawada Y, Ayabe SI (2005). Multiple mutagenesis of P450 isoflavonoid synthase reveals a key active-site residue. Biochem Biophys Res Commun.

[33] Baylon JL, Lenov IL, Sligar SG, Tajkhorshid E (2013). Characterizing the membrane-bound state of cytochrome P450 3A4: structure, depth of insertion, and orientation. J Am Chem Soc.

[34] Ignea C, Trikka FA, Nikolaidis AK, Georgantea P, Ioannou E, Loupassaki S (2015). Efficient diterpene production in yeast by engineering Erg20p into a geranylgeranyl diphosphate synthase. Metab Eng.

[35] Wong J, de Rond T, d’Espaux L, van der Horst C, Dev I, Rios-Solis L (2018). High-titer production of lathyrane diterpenoids from sugar by engineered *Saccharomyces cerevisiae*. Metab Eng.

[36] Laursen T, Borch J, Knudsen C, Bavishi K, Torta F, Martens HJ (2016). Characterization of a dynamic metabolon producing the defense compound dhurrin in sorghum. Science.

[37] Neilson EH, Goodger JQD, Woodrow IE, Møller BL (2013). Plant chemical defense: at what cost?. Trends Plant Sci.

[38] Pettersen EF, Goddard TD, Huang CC, Couch GS, Greenblatt DM, Meng EC (2004). UCSF Chimera—a visualization system for exploratory research and analysis. J Comput Chem.

[39] Eswar N, Webb B, Marti-Renom M a, Madhusudhan MS, Eramian D, Shen M-Y, et al. Comparative protein structure modeling using MODELLER. Curr Protoc Protein Sci. 2007;Chapter 2:Unit 2.9. 10.1002/0471140864.ps0209s50 18429317

[40] Nour-Eldin HH, Hansen BG, Nørholm MHH, Jensen JK, Halkier BA (2006). Advancing uracil-excision based cloning towards an ideal technique for cloning PCR fragments. Nucleic Acids Res.

[41] Genee HJ, Bonde MT, Bagger FO, Jespersen JB, Sommer MOA, Wernersson R (2015). Software-supported user cloning strategies for site-directed mutagenesis and DNA assembly. ACS Synth Biol..

[42] Mikkelsen MD, Buron LD, Salomonsen B, Olsen CE, Hansen BG, Mortensen UH (2012). Microbial production of indolylglucosinolate through engineering of a multi-gene pathway in a versatile yeast expression platform. Metab Eng.

[43] Gietz RD, Woods RA. Transformation of yeast by lithium acetate/single-stranded carrier DNA/polyethylene glycol method. In: Methods Enzymol. 2002. p. 87–96. 10.1016/s0076-6879(02)50957-5 12073338

[44] Nielsen MT, Ranberg JA, Christensen U, Christensen HB, Harrison SJ, Olsen CE (2014). Microbial synthesis of the forskolin precursor manoyl oxide in an enantiomerically pure form. Appl Environ Microbiol.

