# Supplementary material for: Engineering of CYP76AH15 can improve activity and specificity towards forskolin biosynthesis in yeast
Source: Microb Cell Fact. 2018 Nov 19;17:181. doi: 10.1186/s12934-018-1027-3 (PMC6240942; doi:10.1186/s12934-018-1027-3)
Supplement: Supplementary file 1 — Additional file 1. Additional tables and figures. [file 12934_2018_1027_MOESM1_ESM.docx]

**Additional data**

**Content**

Additional Table S1

Additional Table S2

Additional Table S3

Additional Figure S1

Additional Figure S2

Additional Figure S3

References

**Table S1**: SRS variants tested in yeast with diterpene synthases producing 13*R*-manoyl oxide (MO) and miltiadiene (MILT).

| Variant | SRS site | MO | MILT |
| --- | --- | --- | --- |
| A99I | 1 | APP | LA |
| A100V | 1 | WTP | LA |
| G104D | 1 | WTP | WTP |
| V207T | 2 | WTP | LA |
| S235G | 3 | WTP | WTP |
| Y236F | 3 | WTP | WTP |
| S235G Y236F | 3 | APP | LA |
| G362V | 5 | APP | NA |
| L366F | 5 | APP | LA |
| G362V L366F | 5 | APP | NA |
| L366E | 5 | APP | LA |
| 473DDP::EL | 6 | APP | LA |
| F476T | 5 | WTP | APP |
| L478M | 6 | WTP | APP |
| L487I | 6 | WTP | APP |
| L478A | 6 | WTP | APP |
| Combinatorial |  |  |  |
| A99I S235G Y236F | 1+3 | APP | LA |
| A99I L366F | 1+5 | APP | NA |
| A99I L366E | 1+5 | NA | NA |
| S235G Y236F L366F | 3+5 | APP | WTP |
| S235G Y236F L366E | 3+5 | APP | LA |
| A99I S235G Y236F L366F | 1+3+5 | APP | NA |
| A99I S235G Y236F L366E | 1+3+5 | NA | NA |
|  |  |  |  |

APP: altered product profile, WTP: wild type profile, LA: lowered activity, NA: not active.

**Table S2:** Genes utilized in this work.

| Gene name | Type | Origin | Genbank accession nr. | Reference |
| --- | --- | --- | --- | --- |
| CYP76AH8 | CYP | *Coleus forskohlii* | KT382348 | Pateraki et al., 2017 |
| CYP76AH11 | CYP | *Coleus forskohlii* | KT382349 | Pateraki et al., 2017 |
| CYP76AH15 | CYP | *Coleus forskohlii* | KT382358 | Pateraki et al., 2017 |
| CYP76AH16 | CYP | *Coleus forskohlii* | KT382359 | Pateraki et al., 2017 |
| CYP76AH17 | CYP | *Coleus forskohlii* | KT382360 | Pateraki et al., 2017 |
| *Cf*POR | Cytochrome P450 reductase | *Coleus forskohlii* | KX151181 | Pateraki et al., 2017 |
| *Cf*TPS1 | Class II diTPS | *Coleus forskohlii* | KF444506 | Pateraki et al., 2014 |
| *Cf*TPS2 | Class II diTPS | *Coleus forskohlii* | KF444507 | Pateraki et al., 2014 |
| *Cf*TPS3 | Class I diTPS | *Coleus forskohlii* | KF444508 | Pateraki et al., 2014 |
| *Ssp*GGPPS7 | GGPPS | *Synechococcus sp.* | ABC98596 | Forman et al., 2017 |

**Table S3**: Primer list

| Name | Sequence | Gene of interest |
| --- | --- | --- |
| Mutagenesis primers - CYP76AH15 | **(Mutagenesis area marked with red)** |  |
| CYP76AH15_G104D_5 | ACGACCAGAUCTCCATCGGGTTTCTGCC | CYP76AH15 |
| CYP76AH15_G104D_3 | ATCTGGTCGUGGTCGCACGCTTGC | CYP76AH15 |
| CYP76AH15_S235G_5 | AATGGCUACTTTGGAAGACTGCTCAAGTTAA | CYP76AH15 |
| CYP76AH15_S235G_3 | AGCCATUAGCTCTACGCTTGATCCCCTGAAG | CYP76AH15 |
| CYP76AH15_L366F_5 | AGGCAGGGAGUGATCAAGTTGTGAATGGATACCTG | CYP76AH15 |
| CYP76AH15_L366F_3 | ACTCCCTGCCUTGCGAGGAAACAGAAGAGG | CYP76AH15 |
| CYP76AH15_G362V_5 | ACCCTCCCGUCCCTCTTCTGCTTCCTCG | CYP76AH15 |
| CYP76AH15_G362V_3 | ACGGGAGGGUGAAGTCGGAGCACTTCTTTGATCA | CYP76AH15 |
| CYP76AH15_G362V_L366F_5 | ACCCTCCCGUCCCTCTTCTGTTTCCTCG | CYP76AH15 |
| CYP76AH15_G362V_L366F_3 | ACGGGAGGGUGAAGTCGGAGCACTTCTTTGATCA | CYP76AH15 |
| CYP76AH15_473DDP::EL_5 | AGAGCTCUTCGGCTTGGCCATCCGCCGT | CYP76AH15 |
| CYP76AH15_473DDP::EL_3 | AGAGCTCUCCCTTGTGATCCGCATCACCTGCT | CYP76AH15 |
| CYP76AH15_L366E_5 | AGGCAGGGAGUGATCAAGTTGTGAATGGATACCTG | CYP76AH15 |
| CYP76AH15_L366E_3 | ACTCCCTGCCUTGCGAGGTTCCAGAAGAGG | CYP76AH15 |
| CYP76AH15_A99I_5 | ACAGATUGCGCAAGCGTGCGGCCAC | CYP76AH15 |
| CYP76AH15_A99I_3 | AATCTGUGCGATGGTTCTCCCGGAAAACACTTGGCC | CYP76AH15 |
| CYP76AH15_A100V_5 | AGGCGGUGCAAGCGTGCG | CYP76AH15 |
| CYP76AH15_A100V_3 | ACCGCCUGTGCGATGGTTCTCCC | CYP76AH15 |
| CYP76AH15_V207T_5 | ACCATTGUGGGTGAGCCTAATTTCGCTGACTA | CYP76AH15 |
| CYP76AH15_V207T_3 | ACAATGGUGGCCACCCCCTCGATGACC | CYP76AH15 |
| CYP76AH15_L478M_5 | ATGGCCAUCCGCCGTGCAACTCCTCTC | CYP76AH15 |
| CYP76AH15_L478M_3 | ATGGCCAUGCCGAAGGGGTCGTCTCC | CYP76AH15 |
| CYP76AH15_L478I_5 | ATCGCCAUCCGCCGTGCAACTCCTCTC | CYP76AH15 |
| CYP76AH15_L478I_3 | ATGGCGAUGCCGAAGGGGTCGTCTCC | CYP76AH15 |
| CYP76AH15_L478A_5 | ACCCCTUCGGCGCGGCCATCC | CYP76AH15 |
| CYP76AH15_L478A_3 | AAGGGGUCGTCTCCCTTGTGATCCGCATCAC | CYP76AH15 |
| CYP76AH15_F476T_5 | ACCGGCUTGGCCATCCGCC | CYP76AH15 |
| CYP76AH15_F476T_3 | AGCCGGUGGGGTCGTCTCCCTTGTG | CYP76AH15 |
| CYP76AH15_Y236F_5 | AGCTTCTTUGGAAGACTGCTCAAGTTAATGGAG | CYP76AH15 |
| CYP76AH15_Y236F_3 | AAAGAAGCUATTAGCTCTACGCTTGATCCCCTG | CYP76AH15 |
| CYP76AH15_S235G_Y236F_5 | ATGGCTTCUTTGGAAGACTGCTCAAGTTAATGG | CYP76AH15 |
| CYP76AH15_S235G_Y236F_3 | AGAAGCCAUTAGCTCTACGCTTGATCCCCTG | CYP76AH15 |
|  |  |  |
| Cloning into yeast vectors |  |  |
| CYP76AH15_EV_5 | ATCAACGGGUAAAAATGGAAACCATGACTCTTCTCCTCC | CYP76AH15 |
| CYP76AH15_EV_3 | CGTGCGAUTCATGGCTTAAGTGGAATGATCCTG | CYP76AH15 |

**Fig. S1.** Multiple sequence alignment of *Rattus norvegicus* RnCYP2A1, *Coleus forskohlii* CfCYP76AH8, CfCYP76AH15, *Hyoscyamus muticus* HmCYP71D55, *Thapsia villosa* TvCYP71AJ6 for SRS identification. Highlighted areas indicate identified SRS regions for RnCYP2A1 (green) by Gottoh 1997, HmCYP71D55 (red) by Takahashi *et al.*, 2007 and TvCYP71AJ6 (purple) by Dueholm et *al.*, 2015. Determined SRS regions in CfCYP76AH15 and CfCYP76AH8 are marked in blue squares.

**Fig. S2**. SRS alignment of CYP76AH8, CYP76AH15 and CYP720B1. Stars indicate sites chosen in Ignea et al., 2014 for mutagenesis.

**Fig. S3**. Chemical structures and GC-MS spectra of analyzed compounds.

References

Pateraki I, Andersen-Ranberg J, Jensen NB, Wubshet SG, Heskes AM, Forman V, et al. Total biosynthesis of the cyclic AMP booster forskolin from Coleus forskohlii. Elife. 2017;6.

Dueholm B, Krieger C, Drew D, Olry A, Kamo T, Taboureau O, et al. Evolution of substrate recognition sites (SRSs) in cytochromes P450 from Apiaceae exemplified by the CYP71AJ subfamily. BMC Evol Biol. 2015;15:122.

Ignea C, Ioannou E, Georgantea P, Loupassaki S, Trikka F a., Kanellis AK, et al. Reconstructing the chemical diversity of labdane-type diterpene biosynthesis in yeast. Metab Eng. 2014;1–13.

Takahashi S, Yeo YS, Zhao Y, O’Maille PE, Greenhagen BT, Noel JP, et al. Functional Characterization of Premnaspirodiene Oxygenase, a Cytochrome P450 Catalyzing Regio- And Stereo-specific Hydroxylations of Diverse Sesquiterpene Substrates. J Biol Chem. 2007;282:31744–54.

Gotoh O. Substrate recognition sites in cytochrome P450 family 2 (CYP2) proteins inferred from comparative analyses of amino acid and coding nucleotide sequences. J Biol Chem. 1992;267:83–90.

Forman V, Callari R, Folly C, Heider H, Hamberger B rn. Production of putative diterpene carboxylic acid intermediates of triptolide in yeast. Molecules. 2017;22.
